# Marrying Past and Present Neuropsychology: Is the Future of the Process-Based Approach Technology-Based?

**DOI:** 10.3389/fpsyg.2020.00361

**Published:** 2020-03-06

**Authors:** Unai Diaz-Orueta, Alberto Blanco-Campal, Melissa Lamar, David J. Libon, Teresa Burke

**Affiliations:** ^1^Department of Psychology, Maynooth University, Maynooth, Ireland; ^2^Department of Psychiatry for the Older Person, Memory Clinic Services, Health Service Executive, Navan and Ardee, Ireland; ^3^Rush Alzheimer’s Disease Center, Rush University Medical Center, Chicago, IL, United States; ^4^New Jersey Institute for Successful Aging, School of Osteopathic Medicine, Rowan University, Stratford, NJ, United States; ^5^School of Psychology, Faculty of Science and Health, Dublin City University, Dublin, Ireland

**Keywords:** process-based approach, neuropsychological assessment, computerized tests, virtual reality, ecological validity

## Abstract

A cognitive assessment strategy that is not limited to examining a set of summary test scores may be more helpful for early detection of emergent illness such as Alzheimer’s disease (AD) and may permit a better understanding of cognitive functions and dysfunctions in those with AD and other dementia disorders. A revisit of the work already undertaken by Kaplan and colleagues using the Boston Process-Approach provides a solid basis for identifying new opportunities to capture data on neurocognitive processes, test-taking strategies and response styles. Thus, this critical review will combine traditional process-based assessment strategies with support provided or offered by newer technologies that have the potential to add value to data collection and interpretation. There is now considerable interest in neuropsychological test administration using computer/digital technology, both in research and in clinical settings. To add value, any computerized version of an existing cognitive test should respect the administration procedure for which normative data were obtained, should be time-saving in terms of scoring and interpretation, and should, we argue, facilitate gathering information about the processes and strategies followed in test completion. This article will offer an overview of the steps needed when implementing computerization of neuropsychological tests using a Process-Based Approach (PBA) to these technology-based adaptations and will discuss further developments in this area by linking it to future technological developments that may be possible in the area of neuropsychological assessment. Additionally, an overview of neuropsychological tests that may benefit from computerization will be presented, together with suggestions on the specific processes, strategies and features that may be captured with the aid of such computerization. Finally, hypotheses on how virtual reality could be an asset for the future of the PBA to neuropsychological assessment will also be discussed.

## Introduction

Apart from differences in the number of cognitive domains assessed, a common feature of many cognitive protocols administered to detect cognitive decline in clinical practice is that interpretation relies almost exclusively on a series of norm-referenced summary scores. Depending on the test, this could be a single or composite score or multiple subscores summarizing putative cognitive domains that are, at best, identified through a statistical process such as factor analysis and, at worst, derived and labeled based on *a priori* assumptions regarding the nature of the cognitive demands of individual tests and subtests. Some test protocols have identified distinct cognitive domain scores via robust statistical analyses and normative data collection (e.g., Repeatable Battery for the Assessment of Neuropsychological Status -RBANS- by Randolph et al. (1998); or the Wechsler IQ tests, although, as detailed by Canivez and Watkins (2016), concerns persist about the structural validity of the WISC factors and the manner in which they were obtained). In other test protocols, scores are combined into subscores or total scores based on less reliable strategies such as the test developer’s conceptualization of neurocognitive domains. While this latter approach may provide adequate domain-specific scores in one study or context, the lack of statistical validation of these constructs makes it difficult to compare them with other studies and/or patient populations.

Many of the cognitive screening tools available for research purposes and clinical practice provide a cut-off score for ease of data interpretation and, in these contexts, an impaired overall score is typically used to detect the clinical syndromes of Mild Cognitive Impairment (MCI) and dementia. This reliance on a limited quantitative method may, however, lead to erroneous clinical interpretations. As advanced by clinicians such as Lezak (1995) and Kaplan (1988), and others, dating back over many decades, an impaired score on any given cognitive test might be attributable to a range of underlying cognitive deficits, the nature of which are hidden within a single (or small group of) index score(s). In order to circumvent this shortcoming and enhance the scope of clinical interpretation, a traditional quantitative analysis of cognitive test performance reliant on a single or small number of summary/cut-off scores can be complemented with the process-based approach (PBA) methodology developed by Kaplan (1988), Kaplan et al. (1991). This approach emphasizes the importance of error analysis and the analysis of task-completion strategies. It focuses on “how” a task is performed as well as “why” the individual has difficulty on the task. As illustrated below, analysis of this nature can assist in early and differential diagnosis of dementia and MCI. The PBA dates back to Edith Kaplan’s work at the Boston Veterans Administration Medical Center, United States (Milberg et al., 1986) and it is derived from combining (often complex) tests proven to be valid in differentiating between individuals with and without brain pathology with tests designed to measure more specific aspects of cognition. In his review and critique of the process approach, Erickson (1995) noted that Kaplan and her colleagues observed, in a systematic way, the problem-solving strategies adopted by their patients, thereby facilitating both a quantitative assessment of performance and a dynamic evaluation of information-processing style.

Although recognizing the merits of the PBA, Erickson (1995) criticized the approach for a lack of rigorous psychometric analysis–although, more recently, efforts have been made to develop norm-based error rates for different age groups and education levels (e.g., Block Design – Joy et al., 2001; WAIS-III Similarities -Lamar et al., 2010). Recent examples of the efficacy of this approach show how qualitative features of performance may assist in diagnosing Alzheimer’s dementia (Cahn et al., 1997), or how the analysis of errors may differentiate between Alzheimer’s, Parkinson’s and Vascular dementia (Jefferson et al., 2002), between Alzheimer’s and Frontotemporal dementia (Thompson et al., 2005), and between MCI and healthy controls (Ashendorf et al., 2008).

With or without employing the PBA methodology to their interpretation, standardized neuropsychological tests have been the object of criticism for their lack of *ecological validity*, defined by Sbordone and Long (1996) as “the functional and predictive relationship between the patient’s performance on a set of neuropsychological tests and the patient’s behavior in a variety of real-world settings (e.g., at home, work, school, community)” (p.16). The significant limitation to predict an individual’s performance in real-life settings is particularly notable for those tests intended to capture the complex array of neurobehavioral and cognitive symptoms resulting from damage to frontal brain systems, referred to as measures of executive functioning (Sbordone and Long, 1996; Wood and Liossi, 2006; Attree et al., 2009; Knight and Titov, 2009; Sbordone, 2010; Sbordone, 2014). As noted by Burgess et al. (2006) and Parsons (2015), this shortcoming is hardly surprising as traditional neuropsychological tests were designed to assess cognitive “constructs” (e.g., cognitive flexibility; response inhibition) without considering their capacity to predict everyday functioning. In this vein, although most “construct driven” standardized neuropsychological measures afford the examiner sound psychometric properties, including reliability and demographic adjusted norms that may yield adequate diagnostic validity, in general, the literature suggests that the capacity of neuropsychological test scores to predict real-world performance is, at best, moderate. Furthermore, many non-cognitive variables such as physical (e.g., motor deficits), behavioral and emotional factors and levels of premorbid functioning are responsible for a sizeable proportion of variance in real-world behavior (Chaytor and Schmitter-Edgecombe, 2003; Chaytor et al., 2006).

Three main factors have the potential to hamper the ecological validity of neuropsychological test performance (Marcotte et al., 2010). The first factor relates to the relatively sterile *testing environment* in which cognitive tests are conducted. This is typically a quiet and distractor-free environment, isolating sensorial modalities and controlling environmental conditions like noise or temperature. While this may facilitate optimal test performance, the extent to which such testing environments capture real-life environmental demands is questionable and may jeopardize the accurate prediction of an individual’s level of function in real-life settings with respect to a particular cognitive domain. The second factor relates to the *limited sample of behavior* captured by standardized neuropsychological tests over a relatively limited time-period compared to the complex gamut of cognitive processes elicited by real-world tasks over more extended periods. The third factor relates to the lack of agreement regarding the *specific cognitive constructs* measured by neuropsychological tests. For example, while some authors consider the Trail Making Test Part B (Reitan and Davidson, 1974) as primarily a measure of “cognitive flexibility,” others describe it as a measure of “visual-perceptual processing speed” or “set switching ability” (Gunstad et al., 2008; Schwab et al., 2008). This lack of consensus makes it difficult to align any particular cognitive test score to an appropriate cognitive skill in a real-world setting (Chaytor and Schmitter-Edgecombe, 2003; Marcotte et al., 2010).

Attempts to improve ecological validity of neuropsychological instruments has focused on two conceptually different, but complementary, approaches; *verisimilitude* and *veridicality*. In general terms, verisimilitude is defined as “the topographical similarity of the data collection method to a task in the free environment” (Franzen, 2000, p.47), which makes reference to the extent to which the cognitive demands of any given neuropsychological test resemble the cognitive demands posed by real-life situations. Veridicality, on the other hand, is “the extent to which test results reflect or can predict phenomena in the open environment” (Franzen and Wilhem, 1996, p.93). Most work has focused on verisimilitude and the development of standardized performance-based tests designed to mimic the cognitive demands of real-life situations but administered in the clinical setting. Examples of such tests and test protocols include the Rivermead Behavioral Memory Test (RBMT; Wilson et al., 1985) for evaluation of episodic memory, the Behavioral Inattention Test (BIT; Wilson et al., 1987) for assessment of visual attention and neglect, the Behavioral Assessment of the Dysexecutive Syndrome (BADS; Wilson et al., 1996) for evaluation of executive functions, the Test of Everyday Attention (TEA: Robertson et al., 1996) for the assessment of visual and auditory attention, and the Naturalistic Action Test (NAT; Schwartz et al., 2003) for assessment of level of independent functioning. While increasing the resemblance of any given test measure to what might be expected in everyday situations increases the perceived face validity of the measure, it does not necessarily increase its ecological validity in terms of veridicality unless empirical evidence demonstrates its relationship with the real life situations they purport to capture and, regrettably, robust evidence to this effect is still lacking (Chaytor and Schmitter-Edgecombe, 2003; Chan et al., 2008).

In an effort to overcome the limitations of the existing performance-based measures administered in the clinical and/or laboratory setting, a constellation of naturalistic “function-led” tasks performed in realistic environments have been developed purporting to offer higher ecological validity (Robertson and Schmitter-Edgecombe, 2017). These tasks are performed in either a real-world setting or in a staged physical setting designed to mimic the real-world environment and include structured rules to allow for objective and standardized scoring. Examples of the settings include vocational environments (i.e., office and college classroom) to complete clerical/secretarial tasks, kitchen environments to complete cooking tasks, grocery store/s and hospital environments to complete a number of errands and home environments to complete a series of household activities. These tasks provide a window of opportunity to observe participants spontaneously engage in compensatory strategies and afford a safe controlled environment mimicking real-life settings. While some of the existing results are promising, a review of the literature suggests that the efficacy of using naturalistic tasks to predict functional status has yet to be clearly demonstrated (Robertson and Schmitter-Edgecombe, 2017). Moreover, the poor accessibility, high cost and lack of standardization of many of these tests, together with the wanting evidence of their superior veridicality over the more affordable performance-based tests administered in the more traditional clinical setting makes them difficult to be widely employed by neuropsychologists in clinical practice.

New methods of assessing cognitive functions have been developed in recent years because of the limitations with traditional neuropsychological tests and the more recently developed performance-based tests administered in realistic environments (Climent and Banterla, 2011. Latest developments in the area of virtual reality (VR) technologies have generated interesting resources for undertaking neuropsychological evaluations. Subject to adequate quality, VR reproduces three-dimensional (3D) worlds in which the individual using the system interacts dynamically with a given environment, feeling immersed in that environment, comparable to experiencing a real-life setting (Climent and Banterla, 2011; Iriarte et al., 2016). Within VR, both clinicians and researchers can administer ecologically relevant stimuli placed in a meaningful and familiar context and, as a result, measure responses and behaviors in a more comprehensive way (provided visual and physical characteristics of items, avatars and characters are of high quality and realistic). Additionally, VR technology allows tester-control over stimuli, distractors and other variables, and any or all of these factors can be adjusted depending on the response features of the individual undergoing assessment – thereby allowing more personalized assessment. Moreover, automatized capture of responses may allow a more precise and detailed analysis of behavior (Rizzo et al., 2004) and provides substantial opportunity to document aspects of behavior not routinely captured in traditional assessment tools.

This paper will now present an overview of the process-based computerized developments for neuropsychological assessment that have occurred over the last 20 years, as well as an overview of recent computer and VR-based developments in neuropsychological assessment. It will also formulate recommendations on how best to merge both the PBA and technological advances for the refinement and improvement of neuropsychological assessment tools for the future.

## Process-Based Approach: Rationale for Computerization

The application of a process-based approach to neuropsychological assessment requires understanding the relevance of capturing test-taking performance and response sequences in order to extract relevant information. Such information may explain underlying cognitive functioning in a more detailed way than that based on the mere consideration of the final product or test scores. In an attempt to demonstrate the utility of this approach, we present an overview of the process-based computerized developments that have occurred for selected neuropsychological assessment tools.

### Clock Drawing Test

The Clock Drawing Test (CDT) has emerged as an effective and clinically useful cognitive screening instrument for a wide range of conditions (Hazan et al., 2018) and it is probably one of the tests that has undergone most extensive research using a PBA. Among the potential clock drawing conditions that might be used in clinical practice, the most popular ones are the command condition (in which the individual is asked to spontaneously draw a clock with all the numbers on it and the hands pointing to a specific time) and the copy condition (in which the individual is asked to copy a clock from a given exemplar). Lessig et al. (2008) found that up to eleven different error types (out of 24 observed error types) were significantly associated with dementia in individuals with ≥5 years of education, and six of these eleven errors (i.e., no hands, missing numbers, inaccurate time setting, number substitutions or repetitions, or refusal to attempt clock drawing) could be combined to identify dementia with 88% specificity and 71% sensitivity. As the authors noted, “these errors require minimal conceptual classification and are easily detected and scored by non-specialists” (Lessig et al., 2008, p. 460). Later, Price et al. (2011) found that introducing the copy version of the CDT together with the command version in the administration of the Montreal Cognitive Assessment (MOCA) (Nasreddine et al., 2005) helped illuminate the nature of the cognitive difficulties underlying errors in the command condition, and supported differential diagnosis between Alzheimer and vascular dementia patients based on the performance comparison between the Command and Copy versions. Specifically, Alzheimer’s patients performed worse in the command version – thus showing problems with semantic and episodic memory – and those with vascular dementia performed worse in the copy version – thus showing greater difficulties in visuoconstructional and executive abilities. Inspired by this idea of complementing individual tasks within the MoCA, our research group developed a PBA version of the MoCA that, among other modifications, recognizes the relevance and complementary nature of including both the command and copy condition of the Clock Drawing (Blanco-Campal et al., 2016; Blanco-Campal et al., 2019).

Despite the considerable advantages of using a PBA with the traditional paper-and-pencil version of the CDT, the way in which examiners use manual scoring systems varies considerably and examiners may overlook subtle but clinically significant features that could signal the presence of an early cognitive impairment. Binaco et al. (2018) propose the use of machine-learning algorithms to establish a relationship between features of performance in the CDT (captured digitally) and the level of cognitive decline. This may be due in part to the fact that machine learning has the capability to automate time-consuming and subjective processes, analyzing data that is difficult for the clinicians to interpret manually and helps in detecting cognitive impairment at an earlier stage than is possible currently (Souillard-Mandar et al., 2016; Binaco et al., 2018). In this sense, the development of a Digital Clock Drawing Test (dCDT) has provided a more sophisticated and granular evaluation of the cognitive processes involved in the CDT. The digital capture of behavior reduces the practical difficulties associated with the manual scoring and interpretation of the CDT, by way of employing a PBA and opened up a larger dataset for consideration using machine learning.

As described by Davis et al. (2017), the data collected with the digital version of the CDT (dCDT) is time-stamped, allowing capture of both the end result (the drawing) and the behavior that produced it. Drawing time, pauses and hesitations in drawing and time spent holding the pen but not drawing, likely reflecting “thinking” time, is recorded and with 12 milliseconds accuracy. Binaco et al. (2018) describe how training a neural network type classifier with hundreds of features from individual clock drawings could achieve an accuracy for differential diagnosis of 70–80% between subtle cognitive impairment, MCI and Alzheimer’s dementia. The added value of a dCDT would be to achieve higher than practitioner level classification accuracies given only clock drawing data in terms of (1) how the clock is drawn, (2) how long it takes to draw each part of the clock (i.e., each construction variable), and (3) where each construction variable is drawn.

One of the studies that most clearly shows how a dCDT can outperform traditional administration and scoring methods was developed by Cohen et al. (2014), in which they recorded cognitive and motor output in 106 participants grouped by age (younger versus older) and affect (euthymic versus un-medicated depressed), and focused on how the clock was drawn rather than focusing on the final product. In this research, total clock drawing to command and copy time to completion was expressed as percent of time spent drawing (“ink time”) versus percent of time not drawing (“think time”), i.e., pauses between pen strokes likely related to unobservable cognitive activity. They found that younger individuals with depression spent a smaller proportion of time “drawing” the clock relative to the time spent not-drawing or “thinking,” compared to older individuals with depression. Furthermore, “think time” correlated negatively with attention/information processing in younger and older individuals with depression. Thus, despite similar overall performance, nuanced dCDT variables of “Ink” and “Think” times differentiated aspects of psychomotor slowing between younger and older individuals with depression.

These measures of times have undergone a further analysis and refinement using machine learning. As Libon et al. (2015) describe, some of the dCDT features or variables include “…intra-component latencies or the time elapsed between clock drawing components (i.e., time between last element drawn followed by the first clock hand); inter-digit latency (i.e., average time between drawing numbers); and quartile drawing time (i.e., total drawing time divided into four equal segments)” (Libon et al. (2015, p.1). These dCDT latency measures have been related to processing speed and executive functioning performance known to be impacted negatively by Multiple Sclerosis (Libon et al., 2015). Later, Piers et al. (2017) found age-related differences in clock-drawing completion time, total number of pen strokes and latencies in higher-order decision-making in both command and copy task variants in a large sample of stroke and dementia-free individuals included in the Framingham Heart Study, suggesting age-related latency differences that could be a reflection of greater demands on working memory and self-monitoring. According to the authors, these measures have potential to serve as biomarkers of dementia syndromes such as AD and other insidious neurodegenerative disorders. Hizel et al. (2019) used dCDT latency measures to study differences between older adults who did and did not undergo total knee replacement surgery, showing that clock-drawing completion time slowed for nearly 25% of the clinical sample, with relatively longer latencies in both time-to-completion and digit placement. In addition to latencies, Lamar et al. (2016) described the clinical utility of observing the process of “anchoring” digits (i.e., putting the cardinal numbers 12, 3, 6, and 9 before any other numbers on the clock face as a clock drawing strategy). Anchoring was associated with more parsimonious drawings such that “anchorers” needed fewer strokes to complete their drawings and they outperformed “non-anchorers” on tests of executive functioning, learning and memory. In terms of neuroimaging correlates, the unprompted (as nobody tells the individual to use it as a drawing strategy) graphomotor organization that represents “anchoring” was associated with “…more sophisticated modular integration involving the ventral (“what”) visuospatial processing stream.” (Lamar et al., 2016 p.301).

When one considers the progress in computerization of the CDT, a test demanding graphomotor abilities, it seems reasonable to expect that the extrapolation to other graphomotor tests such as the Copy Cube task of the MoCA or the Rey-Osterrieth Complex Figure Test (Meyers and Meyers, 1995) is also possible. Features that might be captured and extracted include latencies from one drawing feature to the next, stroke interruptions, “think time” versus “ink time,” sequence and process of completion (which would allow examiners to differentiate between analytic/detail-focused and holistic/configurational drawing strategies), analysis of error subtypes (e.g., pull to stimulus, lines missing, spatial positioning errors, motor perseverations, tremors or segmentation and rotation errors (Diaz-Orueta et al., 2018) or implementation of alternative structured instructions to uncover performance difficulties in certain groups with cognitive difficulties (Kirkwood et al., 2003).

### Trail Making Test

The Trail Making Test (TMT) is a complex cognitive task requiring multidimensional cognitive skills, some of which are of a lower order (such as numerical and alphabetical knowledge required for sequencing, visual scanning and processing speed) and others of a higher order, including the ability to sustain attention and switch flexibly between two cognitive sets (Delis et al., 2001). The TMT has been used extensively for over 75 years. As early as 1958 and due to the inadequacy of using total time to completion, Brown et al. (1958) defended the possibility of developing qualitative methods of errors analysis. This approach of using more than completion time persists. For example, Ashendorf et al. (2008) have shown how a combined error and time algorithm for the TMT-Part B can yield somewhat higher specificity and positive predictive power (to differentiate between normal controls, MCI and AD) than did completion-time or errors alone. Among the error types that have been identified using a PBA in the TMT, Ashendorf (2013) described sequencing errors, which occur when the letter-number alternating set is preserved but the target selected is incorrect (e.g., 1-A-2-B-4 or 1-A-2-C) and set-loss errors, which occur when the alternating set is not maintained and the test taker continues within set (e.g., number-to-number). While sequencing errors are typically interpreted as reflecting working memory interference arising from difficulty managing both aspects of the alternating sequence, set-loss errors are typically interpreted as reflecting either problems with cognitive flexibility or as difficulty remembering or following task instructions.

Recently, Woods et al. (2015, p.3) introduced a computerized version of the TMT (cTMT) that “systematizes TMT administration, reduces the influence of the examiner, automatically corrects errors, equates Trails A and Trails B path lengths, and presents a standardized TMT display throughout the test that is consistent across subjects.” They go on to say that the cTMT “also permits a segment-by-segment analysis of performance and adds a number of new metrics that provide additional insight into the different factors contributing to overall completion time” (p.3). As detailed by Woods et al. (2015), the new metrics derived from the cTMT include: “(1) segment-by-segment measures of performance; (2) separate measures of dwell-time and move-time for each segment and for the test as a whole; (3) separate measures of error incidence, error time, drawing circuitousness, and drawing velocity; and (4) a complete digital record of paths drawn” (Woods et al., 2015, p.25). However, they also report that error rates are higher than in the paper version of the test because of subjects clicking outside the boundary of the circles.

In this regard, a careful consideration of such a computerized version of TMT needs to be made to maximize the added value of performance capture without adding scoring and interpretation difficulties that arise because of the computer interface. In other words, if a computer interface is going to influence performance negatively instead of just tracking performance in a more precise way, staying with the traditional paper-and-pencil version of the test or adapting the computerized task to address the issues at hand should be prioritized. Fortunately, it appears that the developers of the cTMT are aware of its current limitations and the causes of the difficulties; the key first step to refining test development.

Further refinement of a computerized TMT that addresses the current limitations would, we believe, allow us to better capture performance of the whole test completion sequence. Apart from total completion time already recorded in traditional pen-and-paper administrations of the test, between-item latencies (i.e., between numbers in version A and between letters and numbers in version B), sequence and set-loss errors, segment-by-segment measures of performance, drawing speed, comparison between “think-time” and “ink-time” as in the CDT, strokes and tremors (and probably other measures that have not yet been considered) could be captured in a more precise and accurate manner, and hence offer a more detailed profile of cognitive processes underlying TMT performance.

### Verbal Fluency Tasks

The Controlled Oral Word Association Test (COWAT) (Benton and Hamsher, 1978), also known as the verbal fluency test, requires the participant to generate lexical items while simultaneously observing specified rules or restrictions, such as, for example (1) the words must commence with the specified letter (2) proper nouns are not permitted, and (3) grammatical modifications of prior responses cannot be used. It represents a complex task that places substantial demands on executive and supervisory processes, entailing, as it does, the development of search strategies that are based predominantly on lexical representations but it also depends on more fundamental cognitive components such as attention, spelling-ability and word knowledge (Hodges et al., 1991; Lezak, 1995; Blanco-Campal et al., 2019).

However, as recognized by Lamar et al. (2013) and as demonstrated by the recent findings of Vaughan et al. (2016, 2018), inclusion in a test protocol of a semantic fluency task that can then function as a contrast with phonemic fluency has real potential to add value to the screening for MCI and dementia. Previously, Carew et al. (1997) had found that the capacity to use semantic organizational strategies was relatively intact in patients with ischemic vascular dementia (VD) compared to patients with AD. Although they noted no significant differences between VD and AD participants in terms of overall number of words generated on the “animals” semantic fluency task, they concluded that the performance of VD participants was consistent with search-retrieval deficits, while for AD participants degraded semantic knowledge likely underpinned poor performance (Carew et al., 1997). Thus, consideration of both phonemic and semantic fluency, and the discrepancy scores between these tasks can assist differentiating between normal and pathological subtypes of cognitive aging (Lamar et al., 2013; Vaughan et al., 2018).

Based on the review by Diaz-Orueta et al. (2018), it is important from a PBA perspective to (1) record words generated within 15-s epochs (e.g., 0–15, 16–30 s), (2) identify set-loss errors, and (3) register indices for semantic clustering and switching (as proxies for semantic categorization and cognitive flexibility). These indices, they argue, can be compiled for any verbal fluency task included as a part of a cognitive assessment battery. The authors also highlight Troyer et al. (1997) proposition that a scrutiny of clustering and switching scores can elucidate the reasons why an individual performs as they do, and their assertion that these scores are sensitive to the effects of aging and the status of divided attention. In this vein, Fagundo et al. (2008), in an interesting prospective study, observed that emergent AD was better predicted by the qualitative index of clustering than by total or switching scores. However, these indices have not always been deemed useful or reliable. According to Ross et al. (2007), COWAT performance may necessitate word retrieval in a non-routine fashion that requires the ability to inhibit habitual responses and the linked processing interference, “presumably due to a spread of activation across semantic or lexical networks” (p.475). If correct, this would imply that clustering and switching on the COWAT may “not be entirely deliberate, but rather an artifact of a passive (i.e., state-dependent) process” (Ross et al., 2007, p.475). In contrast, Wajman et al. (2018) found that cluster size could help differentiate between healthy controls and dementia groups (mainly, Alzheimer’s and behavioral variant of Frontotemporal dementia), while the switching component showed smaller capacity to differentiate between the clinical groups.

Technology may provide opportunity for clarification on the subtle components of performance in verbal fluency tasks and on the value of switching and clustering as reliable indices of differential performance between normal and pathological aging conditions. For example, Farzanfar et al. (2018) used an automated computational approach to yield switching, mean cluster size and cumulative relatedness indices in individuals with cognitive decline in Parkinson’s disease. These researchers found that automated switching indices showed evidence of concurrent and construct validity, characterized individual difference in advanced PD, and outperformed the experimenter-dependent index in predicting MCI associated to PD. In contrast, mean cluster size did not appear to be a good indicator of the contribution of the semantic memory component. In a similar research approach, Linz et al. (2017), based on transcribed speech samples from older adults, proposed a statistical method employing distributional semantics. This approach was designed to overcome the problem posed by the fact that the definition of a semantic cluster is usually based on hand-made taxonomies and test performance is typically evaluated manually. With a sample of 100 French older adults, 47 healthy and 53 diagnosed with MCI, they “used distributional semantic models to cluster words in each sample and compare performance with a taxonomic baseline approach in a realistic classification task” (Linz et al., 2017, p.1). The models outperformed the baseline approach but examination of semantic chaining versus semantic clustering models yielded no results favoring either one or the other. Using Troyer et al. (1997) approach, they showed, for example, that there is just one category for water animals within which both frog and dolphin appear. Thus, frog and dolphin fall within the same semantic cluster of animals but this may not capture well the differences between these exemplars. Thus, the researchers concluded that “..human-made taxonomies are error prone and likely to be incomplete” (p.2).

In a parallel study, the same research group (König et al., 2017) introduced a technology for speech analyses for the assessment of cognitive impairment in older adults. They recorded 165 participants with subjective cognitive impairment (SCI), MCI, AD and mixed dementia with a mobile application while they performed a range of verbal tasks, including verbal fluency. By means of the extraction of vocal markers that were tested for their ability to differentiate between cognitive conditions based on machine learning methods, they found that verbal fluency could distinguish between SCI and AD, and between SCI and mixed dementia, in both cases with a 92% accuracy; and between SCI and MCI, and between MCI and AD, in both cases with a 86% accuracy. In a subsequent study, Tröger et al. (2018) developed this technology further by means of a machine learning classifier trained on the French Dem@Care corpus, which, utilizing only vocal features of recorded speech of participants performing three variants of story-telling (positive, negative, and episodic story), could reach a classification accuracy of 89% to distinguish between AD and healthy controls. The developers hypothesized that they could achieve around 99% sensitivity and over 90% specificity by further optimizing reliable detection of silence/sounding, and voiced/unvoiced segments and by the inclusion of Automated Speech Recognition, semantic and pragmatic features extraction tools. Moreover, this system could be used to monitor deterioration over time by within-subject comparison of test results, and, as Tröger et al. (2018) state, without the need for developing parallel test versions for retesting.

Other possible extensions of this type of automatic speech recognition system include the integration of word prototypicality. The Battig and Montague (1969) category norms, as described by Van Overschelde et al. (2004, p. 289), “have been an invaluable tool for researchers in many fields, with a recent literature search revealing their use in over 1600 projects published in more than 200 different journals.” Van Overschelde et al. (2004) expanded these norms to include new categories and new measures, most notably latencies for the generated responses. These 2004 norms are based on response proportions, as they are more informative for researchers because the top responses in different categories can vary dramatically in the proportion of participants giving that response (e.g., water was generated by almost 100% of the participants and eagle was generated by only 47% of the participants, even though both responses were the most common response for their category –“a Liquid” and “a Bird,” respectively). Van Overschelde et al. (2004) also provide the cutoff of 0.05 (5%) for low frequency responses.

In summary, verbal fluency tasks could benefit substantially from an automated collection, processing and detection of performance patterns by means of technological developments; especially if one considers the amount of information that could, as a result of these advances, be collected and made available for further analysis and interpretation. Such developments would permit the clinician to consider readily and easily not just total scores, discrepancy scores and other PBA indices (e.g., partition into 15-s intervals, clustering, switching) that are currently available only through significant clinician effort. Moreover, if an automated speech recognition system could register and distinguish performance profiles based on frequency or prototypicality of individual exemplars within a given category, this would represent, we believe, a significant improvement over the current quantified approach based on the total number of exemplars (regardless of the prototypicality) for an earlier detection and differential diagnosis of neurodegenerative diseases.

### Block Design

The Block Design Test (BD) is a subtest from the Wechsler Intelligence Scale corpus that requires respondents to assemble red, white or red-and-white blocks in three-dimensional space based on presentation of a two-dimensional stimulus card (Swenson et al., 2013). Successful completion of test items requires specific analytic (mental segmentation into individual blocks) and synthetic (see the whole design) problem solving abilities. In order to evaluate performance properly, Kaplan et al. (1991) suggested expanding the BD testing and scoring procedures, such as providing more blocks than specified in the instructions (to detect spatial and executive impairment), drawing flow charts (of each block move), recording error subtypes (rotational errors, broken configuration, orientation errors, perseverations), stimulus bound behavior (e.g., placing blocks directly on the stimulus booklet), response latency, and start position. Using aspects of these approaches, Troyer et al. (1994) identified single and multi-block rotation errors as quite common with increasing age, while Swenson et al. (2013) documented a combination of stimulus bound errors, broken configurations, and psychomotor slowing as the most frequent in those with a dementia diagnosis.

Given the limitations of a manually produced flow-diagram to register all block moves in a timely manner, and to account for performance that is conducted in a three dimensional space, technology could assist in providing clinicians with higher accuracy levels to register respondents’ performance. By way of example, Cha et al. (2009), used automated multi-level analysis with a ceiling-mounted camera that captured an overhead video recording of the respondent’s BD test performance on a table surface, and showed how machine-learning based automated classification can lead to a frame-level overview of the status of the block model and the individual’s actions in undertaking the tasks. Moreover, they also presented an algorithm for sequence-comparison that is able to classify individual problem-solving strategy in relation to a database of simulated strategies.

The question for clinicians and neuropsychologists would be whether all or any of this data capture and data analysis is necessary. Will the sequence-comparison algorithm for classification of performance in BD outperform a trained clinician’s “eye” in the use of the BD test? Is the real added value of this approach the ability to record performance and capture the whole sequence of block completion moves without the need for tedious and difficult hand-made flow charts as were proposed by Kaplan et al. (1991)? Is that sufficient justification for the additional data capture? These and other questions require answers as neuropsychology moves to incorporate such technology into its day-to-day assessments.

To be useful clinically, integration of technological advances for a constructional test like the BD should, we believe, utilize a technological device or system that allows the registration of the full sequence of performance, thus permitting the capture and documentation of different errors types (e.g., stimulus bound, broken configurations, rotations) and aspects of performance (completion times, think-time, psychomotor slowing, etc.) in a way that adds value and outperforms the clinicians’ ability to capture these same data by hand. Speculatively, haptic VR systems or Augmented Reality systems allowing use of real blocks while capturing performance more accurately by means of different types of cameras or sensors could represent an interesting addition. These options would, however, require further in-depth research, and a cost-benefit analysis on the utility of these types of systems when compared with the traditional test protocol.

### Digit Span

Digit Span (DS) refers to a psychological test(s) that assesses auditory span and working memory. It requires individuals to keep in mind and then recall increasingly lengthy series of digits for a short time period. As far back as 1991, Kaplan et al., reflecting their PBA to assessment, suggested that a potentially informative scoring procedure for DS is to tally the number of digits correctly recalled at the highest span correct, rather than the number of successful trials completed (as in WAIS and other test protocols). Additionally, two different processes could be registered, as items recalled in any order, not necessarily serial order, would reflect features of working memory characterized primarily by short-term or immediate storage and rehearsal mechanisms, while items recalled in serial order would reflect more complex features of working memory that require mental manipulation (Lamar et al., 2013).

In recent years, Woods et al. (2011) developed a computerized error analysis in the DS, identifying two general type of errors:

(1) *Item errors*: when the presented and the reported digit string contains different digits, (1a) omission: when the individual omits a digit(s), but the remaining digits are reported in correct order; (1b) additions or intrusions: responses characterized by the spontaneous inclusion of more digits than originally presented; (1c) substitution: a wrong number replaces a number of the original string, without lengthening the original string; also, additions can be considered automatized if the added number is sequential (e.g., 7-4-5-3 repeated as 7-3-4-5-6), which is suggestive of an executive control or frontal systems problem.
(2) *Order errors*: where the reported list contains all of the presented digits but in incorrect order; (2a) transposition errors: when an individual transposes two digits (e.g., “3–4–5–6” is reported as “3–5–4–6”; and (2b) permutation errors: when digit order is incorrect for reasons other than transpositions (e.g., “3–4–5–6” is recalled as “3–6–4–5”).

According to Woods et al. (2011), computerized analysis of error-patterns improves test sensitivity, as it improves the accuracy of the assessment of list length and serial-position effects, error analysis and the detection of malingering.

Given these developments, we believe that although the scoring of individual digit span tests may not be a daily challenge for clinicians, studies with large samples and extraction of data from large databases on DS performance (both forward and backward) could benefit from computerization, as it could assist in the identification of differential profiles that may support performance-based differential diagnosis. Similarly, the Corsi Block–Tapping Test (Corsi, 1972) could, we believe, benefit from computerization [as already described by Berch et al. (1998)] but with the addition of a more detailed computerized analysis of error patterns as in the case of DS.

### Other Tests: Additional Evidence on the Relevance of Error Pattern Analysis

Study of other tests and protocols using a PBA has led to increased awareness of the benefits of such an approach to uncover underlying cognitive deficits by means of the study of processes and strategies used to complete test performance. Studies of error patterns in the Tower test of the Delis- Kaplan Executive Function System (DKEFS) (Kaplan et al., 1991), for example, have shown that individuals with lesions in the lateral prefrontal cortex showed longer completion time and higher rates of rule violations (the latter being a measure with an 83% sensitivity and 100% specificity to detect lesions) (Yochim et al., 2009). Separately, for the Rey Auditory Verbal Learning Test (Rey, 1958), analysis of errors and, more specifically, of intrusion errors, has shown that intrusions appear to reflect subtle cognitive change or compromise that may help predict progression from normal aging to MCI or mild dementia (Thomas et al., 2018). Finally, error analysis in the Similarities test (Giovannetti et al., 2002) has shown that, for AD patients, increasing out-of-set errors (i.e., responses that do not relate both items that need to be compared, e.g., dog-lion: “a lion can eat a dog”) and decreasing in-set errors (i.e., vague relation between the items, e.g., dog-lion: “they can be trained”) was significantly associated with increasing numbers of vascular comorbidities (Lamar et al., 2010). Conversely, a higher pattern of differentiation errors (i.e., “the lion lives in the jungle and the dog in the city”) is more specific of frontal patients, thus showing a good discriminative validity between behavior variant of Frontotemporal Dementia and AD (Garcin et al., 2018).

With all of this evidence, the question that arises is whether we could take advantage of computer-based technologies to increase and improve error analysis; more specifically, to identify disease-specific error patterns and behaviors in a more accurate way than can be achieved currently by means of clinicians’ manually executed scoring. In relation to this question, we would argue that VR technology might allow neuropsychology to reach this next level. Thus, the next section of this paper will present an overview of the relevance of VR-based developments and what they have offered so far in the field of clinical neuropsychological assessment. We then introduce the discussion on how to merge the best of the PBA to neuropsychological assessment with the best advantages that VR technology can offer.

## Virtual Reality Developments and Measures: What They Can Add

Although development of VR technology was initially modest and costs were high (Tarr and Warren, 2002), VR technologies are now readily accessible for use in research and clinical contexts. Today, any gaming computer has the potential to show an immersive, interactive virtual environment, and at reasonable cost. As Riva et al. (2004) show, VR provides a new paradigm of human-computer interaction wherein users are not mere external observers of images on a computer screen, but, rather, active participants inside a computer-generated virtual 3D world. Rizzo et al. (2004) identify some advantages of VR in neuropsychological tools. Among those, we find the possibility to show dynamic and interactive 3D stimuli systematically within a virtual environment (impossible using other means), the ability to create an evaluation environment with greater ecological validity, immediate feedback in a variety of forms and sensorial modalities and, above all, the ability to capture test performance completely (for later data analysis) in a safe evaluation environment. Rizzo et al. (2004) conclude that although VR is not the panacea for all types of behavioral analysis, it represents a great opportunity in terms of usability and usefulness in the neuropsychology arena. However, a tendency has been found to search for correlations with classical evaluation methods, thus leading to mere replications into a digital environment of classical paper-and-pencil tests (Elkind et al., 2001; Ku et al., 2003).

We posit that a relative reductionist approach to VR assessment with a focus on simply replicating paper-and-pencil tests without adding a PBA to administration, scoring or interpretation would not add significant value to neuropsychological assessment. In this vein, Wild et al. (2008) noted that the primary disadvantage of computerized cognitive tests (to date) is that they fail to provide the richness of data than can be derived from a full neuropsychological examination, but they acknowledge that this is not their intent. If VR developments were to focus only on developing a small number of precise quantitative measurements of performance, such as is currently available on traditional pen-and-paper (and many computerized) tests, we would argue that this would be a “costly” exercise of merely replicating the problems of the classic and more affordable neuropsychological tests.

Early virtual environments, such as those using the Wisconsin Cards Sorting Test paradigm, as described by Parsons (2015, p.8), “attempted to build upon the construct-driven neuropsychological assessments found in traditional paper-and-pencil assessments” – but made little attempt to extract additional data. Some of them, like the virtual building described by Pugnetti et al. (1995, 1998) had a heavy reliance on navigating through a building and that navigational component – with which individuals were unfamiliar – may have had a confounding impact on the results. In contrast, the virtual beach used in the Virtual Reality Look for a Match (VRLFAM) (Elkind et al., 2001), modeled on the WCST, was shown to have good convergent validity, with almost all the performance scales being directed related to the original paper-and-pencil task, however, it appeared to do little by way of ecological validity.

**FIGURE 1:**
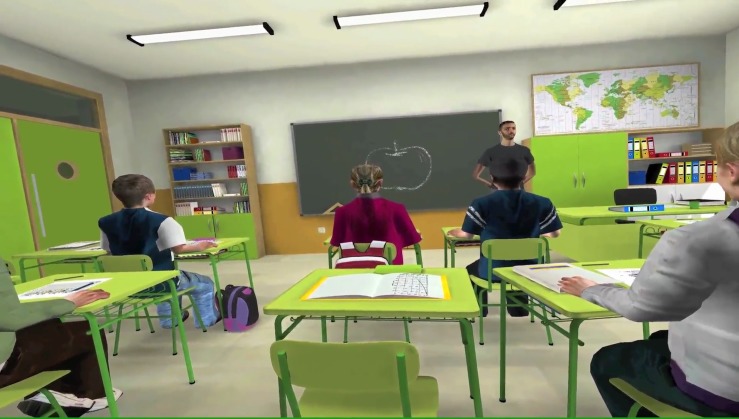
Screenshot of AULA test.

Some virtual environments include the addition of ecologically valid visual and auditory distractors (e.g., someone knocking at the door, a car driving by) to construct-driven tasks (e.g., Stroop or continuous performance tasks). Developments like the Virtual Apartment (Henry et al., 2012), or the Virtual Classroom (Rizzo et al., 2004) enter into this category, as does AULA (see Figure 1; Diaz-Orueta et al., 2014; Iriarte et al., 2016), which is, to the best of our knowledge, the only Virtual Classroom environment that has undergone an extensive normative study with close to 1,300 children and adolescents from 6 to 16 years-old (Iriarte et al., 2016). Although many of these tests have not previously been described as process-based, they have typically tried to integrate data collection that mirrors what could be considered a “process-based” approach to assessment. More specially, these tests have included measures that go beyond a narrow focus on overall test scores. Considering AULA as an example, differentiation of error subtypes, errors by sensorial modality, errors in presence versus absence of distractors, errors in “no-go” versus “go” tasks, body movement tracking, and a particular measure that is referred to as “quality of attention focus” (intended to provide information on whether user’s performance was dependent on internal or external distractors), can be considered, at least to some extent, a test that uses a PBA, with the caveat of presenting data in pure quantitative terms.

**FIGURE 2:**
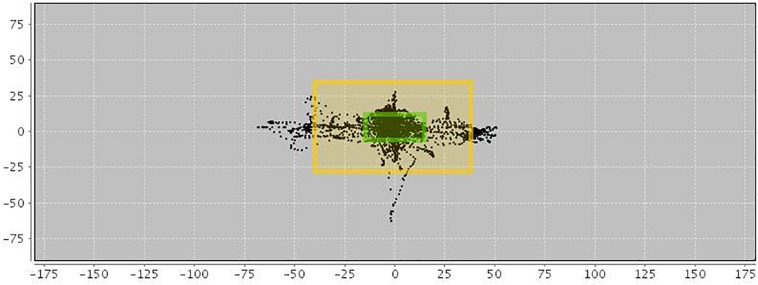
Motor activity in AULA virtual classroom. Black spots within the inner frame indicate a full view of the blackboard. Moves between the inner and outer frames show that the blackboard (and visual stimuli appearing on it) are still visible despite the patient’s moves and deviations. Black spots outside the outer frame show that the blackboard is out of sight.

**FIGURE 3:**
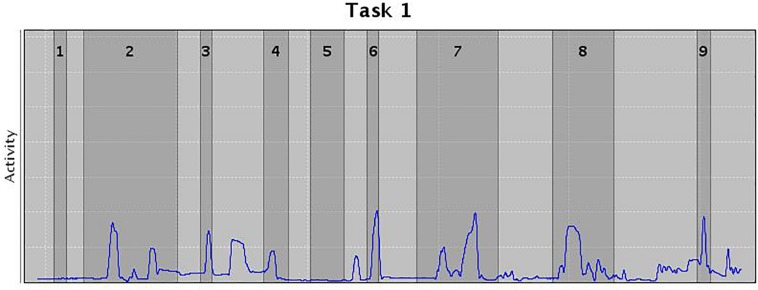
Movements in the presence (dark gray) or absence (light gray) of distractors. Width of the columns indicate the length of the distractor, and peaks the movement wideness.

In addition, an AULA measure of deviation of attention focus shows the amount of time spent by the child with the virtual blackboard out of their sight (see Figure 2), and also provides a report on the co-occurrence of particular head movements in the presence versus the absence of distractors. This measure offers additional information on the nature of attention processes based on the data captured by the head movement tracker of the AULA system (see Figure 3), which, again, might be labeled as using a PBA. Despite the potential advance provided by these types of tests when compared to subjective rating scales of inattention, standardized paper-and-pencil tests or even computerized tests of attention, there is, as Parsons (2015) states, “a need for virtual environment measures that do more than correlate with traditional construct driven measures” (Parsons, 2015, p. 8). In other words, there is a need for VR environments that find a proper balance between respecting the measured constructs and evaluating real-world functions, so that they are ultimately able to predict functioning in a real-life situation.

While research with AULA has focused primarily on individuals with ADHD, other studies using Virtual Classroom environments have shown that VR-based neuropsychological assessment has significant advantages over paper-and-pencil counterparts to evaluate a number of clinical conditions. The study by Nolin et al. (2009) was the first one documented that focused on children with traumatic brain injury (TBI) in order to determine whether virtual environments increase the accuracy and sensitivity of the assessment beyond that obtained with classic computerized flat-screen, two dimensional, continuous performance tests (CPTs). The Nolin group’s later research with adolescents suffering from sports concussions (Nolin et al., 2012), Gilboa et al. (2011) study with patients with neurofibromatosis type 1 (NF1) and Gilboa et al. (2015) study that compared children with acquired brain injury and controls, are studies that demonstrate the VR classroom environment as a useful neuropsychological tool for clinical conditions beyond ADHD. Separately, Parsons and Carlew (2016) focused on autism spectrum disorders (ASD) as a target for VR-based neuropsychological assessment, and found that, under distracting conditions, individuals with ASD performed significantly more poorly than neurotypical controls on the Virtual Classroom Bimodal Stroop task, results that were not found when administering non-VR counterparts. VR-based testing made it possible to find that under conditions of distraction, individuals with ASD are compromised in their ability to activate external distractor inhibition despite the fact that their response time may not suffer. Additionally, Nolin et al. (2016) found a strong concurrent validity between their ClinicaVR: Classroom-CPT and the traditional VIGIL-CPT, and, more specifically, strong correlations were observed between correct responses, commission errors and reaction times, and, interestingly, head movements registered in the VR test correlated with most variables of the traditional VIGIL-CPT, showing that constructs measured by both tests (sustained attention, selective attention, and impulsivity/inhibition) were very similar and supporting the usage of VR-based tests as a way to perform neuropsychological assessment in a way that is more close to real life functioning of test respondents.

More recently, Picard et al. (2017) found that VR-based episodic memory assessment (with a virtual urban environment composed of specific areas, where participants had to memorize as many elements as possible –e.g., scenes, details, spatial, and temporal contexts) was able to show differential recall strategies and patterns associated to increasing age, thus uncovering main developmental differences in feature binding abilities in naturalistic events that are very sensitive to age in comparison with a standard memory assessment. Interestingly, these authors point to the need to further study how and why episodic memory develops in naturalistic settings and the need to better understand the different factors that contribute to these changes and to the variability in performance within participants (which necessarily needs to consider qualitative features of performance).

Despite the apparent advantages of VR-based neuropsychological assessment, however, Neguţ et al. (2016) meta-analysis showed that cognitive performance assessed in VR environments is often poorer than performance observed in pen-and-paper or classic computerized testing leading them to conclude that tasks embedded in VR are more difficult and complex. This leaves open the need to evaluate further the relationship between test performance in VR environments and performance in everyday contexts (i.e., ecological validity – *veridicality*). Almost certainly, ecological validity of VR assessment tools will best be achieved through the adoption of an in-depth PBA to VR developments. With that in mind, we now consider what we believe the future trend for neuropsychological assessment should be.

We argue that if we appreciate the limitations of traditional tests (reliance on a single composite score or a small number of subscores, lack of ecological validity, etc.), acknowledge the potential advantages of a PBA to neuropsychological assessment (for a more rigorous capture of test performance), and recognize the potential of new technologies in terms of computerization and VR, we should be able to “marry” all of these approaches to enhance neuropsychological assessments. The crucial challenges are, we believe, whether we can achieve a norm-based PBA to neuropsychological assessment that can be integrated in a computerized, VR environment and whether clinical neuropsychologists will appreciate the value and feasibility of using these new tests and techniques to enhance their clinical practice. Below, we discuss how a PBA to neuropsychological assessment might, as we conceive it, be merged with the latest technologies to improve the ways that neuropsychological assessments can be performed.

## Marriage of Process-Based Approach With Technology: A Proposal for the Future of Neuropsychological Assessment

In this paper, we have attempted to present an overview of some of the classic neuropsychological assessment tools that have already undergone a PBA and that could, we believe, benefit most from a computerization process. Some of these tests have already undergone computerization, outperforming their paper-and-pencil precedent versions in certain aspects (like the dCDT). It is reasonable to anticipate that a similar approach may be feasible and clinically advantageous to develop computerized versions of most tests involving graphomotor abilities. In other cases, there is clear potential to integrate research work for improving the paper-and-pencil versions of tests via enhanced technology (as with the Block Design with cameras for movements and sequence captures), although in these cases the added value of technology usage would require further research and evidence. Additionally, the work developed in machine learning for speech capture and analysis may advance the study of language and verbal fluency indices of performance for an earlier detection of errors and speech patterns indicative of cognitive decline and diverse neurodegenerative processes. In summary, the opportunities afforded by computerization to a PBA to assessment are (1) a more detailed, focused and accurate observation of behavior, (2) tracking and time-stamping of behavior toward solving a task (by recording responses verbatim in tests with verbal answers and capture of the complete behavioral sequence in any kind of constructional or manipulative test; by identifying processes, detecting and classifying errors and behavioral patterns; and by registering latency from moving through different behavioral steps into a sequence), (3) reduction in examiner data capture and scoring error and bias, (4) reduction in testing procedure times (i.e., increase in efficiency), (5) increased specificity of cognitive/behavioral performances, (6) identification of the emergence of endophenotypic profiles, and (6) delineating state (e.g., fluctuating attention to task, affective state, low motivation) and contextual factors that can influence performance scores.

Here, we are still considering a 2D framework, and the advantages offered by computerization seem quite clear. If we move to a 3D framework, the VR-based developments in neuropsychological assessment can, at least potentially, add value by increasing ecological validity of performance testing. As discussed in relation to some of the VR-based neuropsychological tests examined in this paper, the main advantages described by Rizzo et al. (2004) are present, however, it is reasonable to state that the marriage between the work performed by Luria (1976) and Kaplan (1988), on the one hand, and Rizzo et al. (2004), Kane and Parsons (2017) and many others on the other, has not been in the conscious planning and consideration for the future of neuropsychological assessment.

We consider that if VR environments can recreate real-life environments to make assessment more ecologically valid and data capture more thorough and accurate, that data capture should, in order to add maximum value, include an assessment of behavioral sequences, strategies, error patterns and response styles of respondents as is currently undertaken by those clinicians who, to a greater or lesser extent, adopt a PBA to neuropsychological assessment. We acknowledge the reticence of clinicians to abandon the use of traditional “construct driven” standardized paper-and-pencil cognitive tools to measure cognitive performance without solid grounds to do so, and we expect that a barrier toward technology adoption will arise if VR and, indeed, any subsequently developed technologies, fail to improve upon the performance of a clinical neuropsychologist using traditional cognitive tests. That is to say, if VR-based testing falls short of adding real value to the clinical environment and to the accuracy and amount of information obtained in an evaluation session, one must understand and accept the reluctance of neuropsychologists to dispense with “familiar” cognitive measures in order to integrate VR assessment tools into their clinical practice. However, if VR can increase the veridicality in terms of predicting performance of real-world tasks [as described by Spooner and Pachana (2006)], thus increasing the links between the demands of neuropsychological tests and functional performance, function-led VR based tests may complement traditional neuropsychological batteries, as suggested by Parsons (2015).

When discussing the potential of technology-based neuropsychological assessment to outperform traditional tests, it is relevant to consider whether performance of Instrumental Activities of Daily Living (IADLs), such as the measures used by Ashendorf et al. (2018) (e.g., driving, judgment, bill payment, daily living memory, medication instructions…) might be measured with novel computer-based VR tasks. Work by Schultheis et al. (2006) indicates that new driving-performance measures can be derived with VR driving simulations, and they suggest that these indices may be beneficial for evaluating driving capacity following neurological compromise. If a test like the Naturalistic Action Test (Giovannetti et al., 2008) is able to reformulate a model for everyday action impairment in AD (on which omissions, commissions and action additions reflect distinct everyday action deficits), it is very likely that an integration of cognitive and everyday functional assessment could be feasible in a simulated VR environment. The challenge, of course, would be how to develop a computerization process of such detail that allows inserting natural daily living action into the restrictions of a computerized environment without erasing the “naturalistic” component to the actions that are depicted in the virtual environment.

Our position is that the future of neuropsychological assessment is process-based and that this method could be enhanced significantly by the “new technologies” leading to the creation of well balanced “construct driven and function-led” measures. These foreseen resulting measures would serve to bridge the existing gap between measures focusing on replicating real-world environments to the detriment of experimental control over distinct cognitive processes and those focusing solely on the cognitive construct neglecting the ability to predict functional behavior in real-world settings. We posit specifically that VR, together with machine-learning and other regular computerization and technology-driven tracking devices can have a pivotal role in the future of neuropsychological assessment provided that it incorporates the advantages of a PBA into the development of large-scale, norm-based, VR and technology-driven neuropsychological tests. For us, the ideal future scenario would represent a computerized simulation of a daily life environment that measures cognitive functions and activities of daily living in close-to-real-life situations and demands, while tracking behavioral sequences, verbal and non-verbal responses, patterns and error types, and making use of machine-learning and big data analysis for detection of subtle behavioral patterns indicative of early cognitive decline or other neuropsychological conditions. Undoubtedly, the amount of information provided by a system like this would have the potential to assist clinicians in performing more accurate judgments and predictions of a patient’s daily life, including such things as school or work performance, and could, ultimately, support the development of more personalized, individually tailored rehabilitation programs.

## Author Contributions

UD-O completed a literature review and prepared the initial draft of the manuscript. UD-O and AB-C collaborated in developing the review further. ML, DL, and TB contributed additional literature, critical reviews, and comments of the manuscript. All authors were instrumental in formulating the research question(s) and discussed and agreed on their position concerning the topic presented here.

## Conflict of Interest

The authors declare that the research was conducted in the absence of any commercial or financial relationships that could be construed as a potential conflict of interest. The reviewer TD declared a past collaboration with one of the authors UD-O to the handling Editor.
